# Resuming Swallowing and Oral Feeding in Tracheostomized COVID-19 Patients: Experience of a Swiss COVID-Center and Narrative Literature Review

**DOI:** 10.3390/medsci10040057

**Published:** 2022-09-29

**Authors:** Ruben Forni, Etienne Jacot, Giovanni Ruoppolo, Antonio Amitrano, Adam Ogna

**Affiliations:** 1 CREOC Service of Physiotherapy, EOC San Giovanni Hospital, 6500 Bellinzona, Switzerland; 2 Department of Business Economics, Health and Social Care, University of Applied Sciences and Arts of Southern Switzerland, 6928 Manno, Switzerland; 3 ENT Service, EOC San Giovanni Hospital, 6500 Bellinzona, Switzerland; 4 USI (Università della Svizzera italiana), 6900 Lugano, Switzerland; 5 IRCCS San Raffaele-Pisana, 00163 Roma, Italy; 6 ASUGI (Azienda Sanitaria Universitaria Giuliana Isontina), 34148 Trieste, Italy; 7 Respiratory Medicine Service, EOC La Carità Hospital, 6600 Locarno, Switzerland

**Keywords:** swallowing, tracheostomy, dysphagia, TLI, COVID-19, ARDS, rehabilitation, physiotherapy

## Abstract

During the COVID-19 pandemic, percutaneous tracheostomy proved to be an effective option in the management of patients with prolonged periods of intubation. In fact, among other things, it allowed early discharge from ICUs and contributed to reducing overcrowding in intensive care settings, a central and critical point in the COVID pandemic. As a direct consequence, the management and the weaning of frail, tracheostomized and ventilated patients was diverted to sub-intensive or normal hospitalization wards. One central challenge in this setting is the resumption of swallowing and oral feeding, which require interdisciplinary management involving a phoniatrician, ENT, pneumologist, and speech therapist. With this article, we aim to share the experience of a Swiss COVID-19 Center and to draw up a narrative review on the issues concerning the management of the tracheostomy cannula during swallowing resumption, integrating the most recent evidence from the literature with the clinical experiences of the professionals directly involved in the management of tracheostomized COVID-19 patients. In view of the heterogeneity of COVID-19 patients, we believe that the procedures described in the article are applicable to a larger population of patients undergoing tracheostomy weaning.

## 1. Introduction

Tracheostomy and tracheotomy are surgically created openings from the skin of the neck down to the trachea to put the tracheal lumen in direct contact with the external environment. This opening can be permanent (tracheostomy, with the suture of the trachea to the skin) or temporary (tracheotomy), and facilitates ventilation by bypassing the upper airways. The first interventions of this type are described in ancient texts and were mentioned in the fifth century BC in the Corpus Hippocratium. Galen credited Asclepiades, a Roman physician, as the creator of this type of surgery in the first century BC [1,2]. Many centuries later, the surgical technique became more reliable. Jackson, in 1909, standardized the technique and indications of the operation. Of particular interest is his recommendation not to perform tracheotomy in a high position, as it was the chief cause of laryngeal stenosis [3]. In 1985, Ciaglia first described the percutaneous dilatational tracheostomy (PDT), a variant of the tracheotomy technique [4]. Due to the availability of new technologies—among them the use of a bronchoscope control [5]—the PDT technique has significantly developed since then, gradually becoming the standard in intensive care units (ICUs) because it is a quick and safe technique. Furthermore, compared to traditional tracheotomy, PDT has the advantage of being performed in the ICU directly at the patient’s bedside, without requiring an operating room.

Tracheotomy and PDT both offer several advantages in patient management:

- Facilitated ventilation by reducing dead space for breathing.
- Aid in reducing sedation.
- Facilitated care for ventilated patients allowing them to awaken and mobilize.
- Compatibility with speech and oral nutrition.
- Reduction of risk of laryngeal injury due to prolonged intubation.

Tracheotomy, PDT in particular, has shown all its usefulness, even during the ongoing pandemic. In the Swiss Confederation’s COVID-19 wards, PDT has made it possible to ventilate critically ill patients for a long period and to move them from the ICU to the more appropriate post-intensive wards to lighten the pressure on ICUs, opening beds to welcome new patients, and optimizing the management of hospital resources [6].

Despite the wide diffusion of the technique, there is still no consensus about post-tracheotomy management, particularly regarding the weaning from the cannula, the resumption of feeding and the best way to facilitate speech. During the pandemic period, in the absence of guidelines, internal protocols were developed in Switzerland for the weaning process from the tracheotomy. To identify the best timing for tracheotomy closure, in particular, we developed the Tracheo Score Index (TSI) [6] (Table 1).

A TSI score of four made it possible to remove the tracheal cannula (TC). One of the most relevant issues to consider when decannulating the patient was the resumption of oral feeding. In practice, all tracheotomized patients came from the ICU to the ward with the nasogastric tubes (NGT) to ensure feeding. Oral administration of the macronutrient income necessary to avoid catabolism was possible only for patients in whom the evaluation of swallowing function had demonstrated its efficacy and safety. In the other cases, the removal of the SNG was followed by the placement of a percutaneous endoscopic gastrostomy (PEG).

The aim of this article is to define the most appropriate modalities of decannulation and an optimal model of feeding transition from NGT to oral, integrating the most recent evidence from the literature with the clinical experiences of the professionals directly involved in the management of tracheostomized COVID-19 patients.

## 2. Physiological Effects of the Tracheotomy on Swallowing

Breathing and swallowing are critical to survival. Both functions use the same anatomical structures. In the healthy subject, an efficient system of finely coordinated sphincters regulates breathing and swallowing, avoiding dangerous functional overlaps. In young adults, swallowing interrupts exhalation, which resumes immediately after the act of swallowing [7]. In this way the expiratory flow pushes any pharyngeal residual bolus towards the mouth. In elderly subjects and in subjects with respiratory and/or neurological problems, swallowing often interrupts inspiration. In this case, an eventual residual bolus constitutes a serious risk of aspiration [8,9]. In any case, the presence of the tracheal cannula interferes with swallowing.

Although the literature is still contradictory, probably due to the lack of randomized clinical trials able to provide evidence, most studies argue that the presence of TC coincides with the increase in pharyngeal dysphagia and aspiration [10,11]. Swallowing normally takes place within a closed system whose pneumatic balances are altered by the presence of the TC [12,13]. The presence of TC is believed to be related to numerous effects on swallowing: decrease in vertical and anterior rotation movements of the larynx [11,14], compression of the esophagus due to cuff inflation pressure [10], alteration of laryngeal reflexes [15,16], desensitization of the larynx due to the deviation of the airflow through the TC [11], reduction of the cough reflex due to the accumulation of secretions in the supraglottic space [17,18], reduction of subglottic pressure [19,20], disuse atrophy of the laryngeal musculature [14]. Further studies report difficulties in the formation of the bolus, delayed triggering of the pharyngeal phase, increased residues in the pharynx, and silent aspirations as a consequence of the presence of TC [21]. The role of the cannula cuff in inhibiting the laryngeal elevation and anteriorization movements is also the subject of contradictory investigations: in the study conducted by Bonanno et al. [11], only three (7%) of the 43 participants presented the expected laryngeal mobility deficit. A subsequent study [22] examined the movements of the larynx and hyoid bone through a video fluoroscopic analysis of swallowing in three different conditions: presence/absence of TC; swollen and deflated cuff; closed and open cannula. No significant difference in laryngeal movements was detected in all experimental conditions. Several studies have analyzed the relationship between aspiration, and therefore dysphagia, and the presence of TC [17,23,24,25]. Leder [12] analyzed aspiration in a group of subjects undergoing head/neck surgery. From time to time, subjects were asked to swallow with TC, without TC and with the stoma closed by a gauze pad and without TC with the stoma left open. The presence of aspiration was detected by trans-nasal fibreoptic endoscopic evaluation of swallowing (FEES) and trans-stomial FEES. The study showed 100% agreement in the detection of aspiration with the trans-nasal FEES and the trans-stomial FEES. In the subjects showing aspiration, this was present in all the three experimental conditions. Likewise, the subjects who did not aspirate showed a similar behavior in all the conditions. A further, larger study [24] confirmed the absence of a causal relationship between the presence of TC and aspiration. Studies report how swallowing can improve even in the presence of TC [26] and, conversely, the patient can continue to present dysphagia even after decannulation [27]. The presence of TC therefore does not always cause aspiration. In subjects with TC who have aspiration, this is to be related to the morbid state that led to the insertion of the TC rather than the mere presence of the TC itself. However, a relationship has been found between TC and aspiration in elderly subjects. Subjects older than 72.5 years aspire significantly more consistently than younger subjects with similar clinical conditions [14,28,29]. The higher incidence of aspiration in elderly subjects has been related to the reduction of functional reserve and the lower adaptability to stress [30].

The presence of a TC with an insufflated cuff has been widely considered to protect the airways from the passage of a bolus, since the inflation of the cuff is assumed to prevent aspiration. This claim is questionable for several reasons. First of all, because the cuff is placed below the vocal cords, it cannot block aspiration because this has, in fact, already occurred. In fact, the term ‘penetration’ designates the passage of bolus into the airways that does not go beyond the glottis plane, while the term ‘aspiration’ designates the passage of bolus into the airways when it passes the glottis plane [31]. It is evident that when the cuff blocks the bolus, aspiration has already taken place. Further research has also shown that the inflated cuff does not prevent bolus passage into the lower airways [32,33,34]. In fact, an insufflated cuff blocks the immediate fall of the aspirated bolus into the trachea but does not prevent it from slowly seeping through the contact, which is not watertight, between the cuff and the tracheal wall. Nor is it advisable to improve the seal, which in any case is never perfect, by increasing the inflation pressure of the cuff due to the high risk of producing an ischemia of the tracheal wall. The cuff inflation pressure should never exceed 20 mmHg. The intraluminal pressure of the mucous capillaries is between 25–35 mmHg. Therefore, a cuff inflation pressure above these levels would expose the tracheal mucosa to a serious risk of ischemic damage.

The presence of the tracheal cannula diverts the airflow outwards, preventing or significantly reducing the flow of air through the larynx. The absence of airflow leads to the weakening and reduced coordination of the posterior cricoarytenoid muscles [15], which normally regulate the opening of the glottis. Likewise, the lack of airflow crossing the larynx has negative repercussions on the functionality of the adductor musculature of the vocal cords [35]. It is important to emphasize that the resumption of airflow inside the larynx, which occurs with decannulation or with the closure of the cannula, involves the resumption of normal functionality of the opening and closing mechanisms of the vocal cords [16].

One of the main mechanisms that leads to an increase in aspiration risk in patients with tracheotomy is, however, the partial loss of sensitivity of the superior laryngeal nerve, responsible of the sensory part of the laryngeal adduction reflex. In long-term tracheotomized patients, the reflex evocation threshold is doubled. The attenuation of the reflex also involves a weakening of the glottis closure, which inevitably facilitates aspiration. The presence of a tracheostomy which allows air to escape also reduces the expulsive force of the cough, making it ineffective by eliminating its compressive phase.

In the light of these data, albeit in part contradictory, in the clinical practice, it is necessary to consider the possible interferences of TC in the evaluation and treatment procedures, as well as on the management of the cannula during normal feeding activities.

## 3. Evaluation of Dysphagia in the Cannulated Patient

The analysis of the literature allows us to draw some operational indications to assess dysphagia in TC patients and to define their diet. Dysphagia evaluation in a cannulated patient plays an important role not only for weaning from the tracheostomy tube, but also in the clinical management of the patient. While a cannula of any type by itself does not prevent swallowing, it remains important to evaluate the patient’s swallowing function before reintroducing oral feeding.

The evaluation of dysphagia can take place in a clinical and/or instrumental way. The evaluation of dysphagia in a patient with TC requires additional specific procedures.

A clinical evaluation of a patient with TC must take place with the cuff deflated [36] and, if possible, with the cannula closed or with a speaking valve. If it is not possible to close the cannula even temporarily, a clinical evaluation is nevertheless feasible. In cases in which it is not possible to deflate the cuff, a clinical evaluation is unreliable. In these cases, it is necessary to have recourse to instrumental evaluation. The same applies if a cough reflex deficit is suspected, as underlined by Ajemian et al. [37] in 2001 in their pioneering study. The usefulness of evaluating swallowing function even in this phase should be emphasized because it may be possible for the patient to feed orally even in cases where it is not possible to deflate the cuff. This is particularly true in the case of severe neurodegenerative diseases. Even ventilated patients might be safely fed by mouth after careful instrumental evaluation (FEES).

It should be stressed that a clinical evaluation is not reliable in detecting any silent aspirations, i.e., in cases where the passage of bolus in the airways is not followed by cough. Only instrumental techniques can see the passage of a bolus in the airways. For this reason, they are more sensitive and specific in the evaluation of swallowing in patients with TC.

## 4. Breathing and Swallowing Management in COVID-19 Patients

COVID-19 disease can lead to a progressive respiratory distress that requires mechanical ventilation. In two recent reviews on postintubation dysphagia during the COVID-19 pandemic, Frajkova et al. [38] as well as Ceruti et al. [39] highlighted the specific problems that make the consequences of intubation even more serious in such patients, in particular reduced lung function and frequent comorbidities, resulting in an increased risk of mortality and prolonged ICU stay. Data from Ceruti’s revision showed an incidence of dysphagia of 54.8% in patients admitted to the ICU, with a high recovery rate (90%) at 16 days after rehabilitation. A careful evaluation of dysphagia and proper rehabilitation management are therefore mandatory.

On the other end, according to the recommendations on the management of dysphagia in a pandemic era [40,41], it is necessary to use all the prescribed precautions to make the evaluation safer. The bedside examination, when appropriate, and particularly when cough reflex is present, becomes the first-choice evaluation, as it is less dangerous for healthcare workers [42,43]. The Evan’s blue dye test (EBDT) [44] and the later version, the modified Evan’s blue dye test (MEBDT), are used for the clinical evaluation of swallowing in patients with TC. The test and its subsequent modified version are performed by placing boluses colored with methylene blue on the patient’s tongue, followed by THE monitoring of tracheal aspiration in the following 48 hours. The presence of colored secretions reveals the passage of the bolus in the airways. In the modified version, boluses of different consistencies are administered, while the method of detecting any aspirates remains the same.

## 5. The Experience of the “Tracheo” Sub Intensive Care Unit of the La Carità Hospital of Locarno

The Hospital of Locarno belongs to the public hospital network (Ente Ospedaliero Cantonale) of the Southern part of Switzerland (370,000 inhabitants). Since late February 2020, it has been entirely dedicated to the care of COVID-19 patients, with a capacity of 180 beds and an intensive care unit which was expanded from eight to 45 ventilator-equipped beds. One of the internal medicine wards was converted into a sub-intensive care unit (“tracheo unit”) with a capacity of 24 beds, with the aim to early discharge patients from ICUs, to stabilize them and facilitate the achievement of clinical conditions that made it possible to refer the patient to a rehabilitation centre outside the hospital. During the first pandemic wave, between the 20 March and 31 May 2020, 51 patients were transferred from the ICU to the “Tracheo” Unit, 29 of whom were mechanically ventilated by tracheotomy (six females, 23 males; median age 68 years (IQR 60–72; range 39–78)). The experience gained during the first pandemic wave allowed us to refine the internal protocols for the management of tracheostomy weaning.

From 15 November 2020 to 8 February 2021, 48 consecutive subcritical ill COVID-19 patients (13 females, 35 males), median age 69 years (54–83), were transferred from the ICU to the sub intensive care “tracheo” unit. All patients had previously undergone oral-tracheal intubation, while 26 of them had a tracheotomy and were canulated. Three patients were excluded from this case series, one for death and two others for bounce back to the ICU, in one case for heart disease, in the other for bleeding. All patients were followed by a multidisciplinary team and underwent early mobilization, respiratory therapy, and progressive weaning from ventilation. The swallowing functionality of each patient was evaluated by a speech therapist, by means of the GUSS test, supplemented by the blue dye test in patients with tracheal cannula. Out of the 45 patients, 31 (68.9%) were found to be dysphagic (including all the patients with tracheal canula) and underwent intensive rehabilitation (2 h/day). Twenty-six of them recovered oral feeding and underwent canula removal. In the remaining five subjects, a percutaneous gastric tube was positioned before transferal to the rehabilitation facility, due to the persistence of dysphagia, and the cannula was kept for a safer management of secretions

## 6. Discussion

Examination of the available literature supports the importance of a careful examination of patients’ swallowing function in oral feeding resumption. When oral feeding has been deemed safe, the cuff must be kept deflated, and where possible the cannula should be closed or equipped with a speaking valve. In fact, the cuff, as already seen, does not protect against aspiration and weakens one of the body’s natural defense mechanisms, namely coughing. Any bolus leaks from the cannula or the presence of food residues in the tracheal aspirate should prompt the immediate interruption of oral feeding and a new phoniatric and speech therapy evaluation.

An instrumental evaluation of the patient with a tracheal cannula has to answer the following questions:

- Are there salivary stagnations?
- Does the patient have spontaneous swallowing and is the patient able to swallow saliva?
- Are there alterations in morphology and laryngeal motility?
- Are laryngeal sensitivity and cough reflex preserved?
- Is the patient able to take food orally? Of what consistencies?

Among instrumental swallowing examination methods, FEES is recognized as ideal, as it can be carried out at the patient’s bedside, even with limited patient cooperation. It allows an optimal evaluation of saliva and an accurate observation of the larynx, and it can be repeated without exposing the patient to radiation. In the event of massive pooling of saliva or when the patient does not trigger a spontaneous swallowing act within one minute, the examination must be suspended. Otherwise, laryngeal sensitivity is assessed as the next step, evoking the triggering of the laryngeal adduction reflex by touching the hypopharyngeal/epiglottis mucosa. In the event of a valid reflex, as in all other instrumental evaluations of swallowing, we proceed to test the different consistencies (semi-solid, semi-liquid and liquid), evaluating possible penetration into the larynx and the extent of any pooling, as well as the number of swallowing acts necessary for their elimination.

Finally, in the cannulated patient, retrograde trans-tracheostomy evaluation is possible if doubts persist after endoscopic evaluation.

## 7. Conclusions

The evaluation of swallowing function in patients with tracheal cannula, particularly if transferring from intensive care units and previously subjected to orotracheal intubation, is of crucial importance to patient health and for the optimal management of hospital resources. The decannulation and recovery of oral feeding not only exert a positive influence on the clinical course of the patient, due to nutritional and psychological benefits, but also allow a faster discharge of the patient, with significant savings for the health system. In view of the heterogeneity of COVID-19 patients, we believe that the procedures described in the article are applicable to a larger population of patients undergoing tracheostomy weaning.

## Figures and Tables

**Table 1:** Tracheo Score Index (TSI).

1 point	Patient oriented
1 point	Patient can stay 24 h with the artificial nose without ventilation
1 point	Good cough reflex, patient can stay with deflated cuff and speaking valve or artificial nose without any aspiration
1 point	Patient does not need profound tracheal aspirations

## Data Availability

Not applicable.
